# Obituary

**Published:** 1911-11-25

**Authors:** 


### Obituary.

The death is announced of Dr. John Breakey, of the Royal Navy (retired). He was Inspector-General of Hospitals and Fleets, and qualified in 1851, taking the M.D. of the Royal University of Ireland in 1853.

Surgeon-Major Campbell Dykes, of the Indian Medical Service, has died at Ghazipur. He was formerly house physician at Brompton Hospital for Consumption, and house surgeon at University College Hospital, at which he received his medical education. He graduated M.B. Lond. in 1897.

The death is announced of Dr. Stanley Smith, of Wimpole Street, in the sixtieth year of his age. He was physician to the Railway Clearing System Superannuation Fund. Dr. Smith studied at the London Hospital and qualified in 1877; later he took diplomas at Edinburgh, and in 1895 became a member of the Royal College of Physicians of London and M.D. of Durham University.

The death is announced of Mr. Howard Carson Toone, of Devizes, at the early age of thirty-two. Mr. Toone was a student at Charing Cross Hospital and the Royal Dental Hospital, Leicester Square, from which he took his dentist's licentiate in 1904. He was dental surgeon to the military depôt at Devizes, and enjoyed a successful practice in the town until his health failed through pernicious anæmia, from which he had suffered for over two years before his death, which occurred at Bournemouth.

By the death of Dr. James Nairn McDougal, a much-respected physician is lost to Berwickshire. In the village of Coldingham Dr. McDougal was more than a medical practitioner; he took a great and practical interest in all schemes for its welfare. He was the Medical Officer of Health for Coldingham and a J.P. for Berwickshire. Dr. McDougal was already an M.D. Edin. in 1860, and only retired from active practice five years ago. He was a man of liberal education and a great reader.

The death is announced of Dr. John L. Goodlatte Murray, of Egremont, Cheshire, who had held a popular and influential position as a general practitioner in the neighbourhood of Liscard and Egremont. Dr. Murray spent the first part of his medical career in New Zealand, and in New South Wales, where he was surgeon to the Parkes District Hospital and public vaccinator. Dr. Murray studied medicine both in Edinburgh and in Dublin, taking diplomas in both places; he qualified in 1878, and later became a Fellow of the Royal College of Surgeons, Edinburgh, and also took up the D.P.H. of the Irish Colleges. On settling down in Cheshire, Dr. Murray was elected to the Medical Institute of Liverpool, in which he took a very active interest. His death occurred somewhat suddenly from apoplexy.

The death is announced of Dr. John Alfred Coutts, of Upper Berkeley Street, which took place on November 1 at Barnes, in his sixty-first year. Dr. Coutts was a Fellow of the Royal College of Physicians, to which he was elected in 1897, having taken the membership in 1884. He was Senior Exhibitioner at Emmanuel College, Cambridge, from which he graduated M.B. in 1882. As physician to the East London Hospital for Children Dr. Coutts had a wide experience of the diseases of children, in which specialty he practised, and his contributions in this department of medicine were very numerous. He was also formerly Hunterian Professor to the Royal College of Surgeons. Dr. Coutts's comparatively early death from pneumonia following an attack of influenza robs the profession of one of the most prominent authorities on children's diseases.

The death is announced of Dr. Price Prichard Baly, of Westward Ho, North Devon, which occurred suddenly on October 20, in his fiftieth year. He was a student of Queen's College, Birmingham, and qualified in 1888. Dr. Baly held the appointment of medical officer to the Post Office.

The death is announced of Dr. James Murray Lindsay, of Aldershot. He was a well-known authority on mental diseases, and was formerly President of the Medico-Psychological Association. For many years Dr. Lindsay was superintendent of the Derby County Asylum, and had held several appointments at various institutions for the insane. He qualified at Edinburgh in 1859, and later became a Fellow both of the Royal College of Surgeons and the Royal College of Physicians of Edinburgh. In 1893 he delivered the presidential address at the meeting of the Medico-Psychological Association.

Professor Samuel McBean, late Professor of Materia Medica and Therapeutics at the University of Durham College of Medicine at Newcastle, has died at the age of seventy-two. He qualified at Edinburgh, and in 1862 was appointed assistant surgeon to the Royal Naval Hospital, Plymouth, and afterwards assistant surgeon in the Royal Navy to the *Royal Adelaide*. Later he was appointed surgeon-in-charge of the Royal Naval Hospital, Yokohama. In 1867 he served in the expedition up the Niger, and was afterwards appointed surgeon to the Royal Naval Hospital, Chatham. He retired from the Service to Newcastle in 1868.

Dr. Thos. Hy. Stocker Pullin has died at his residence in Sidmouth, Devon, in his eighty-eighth year. Dr. Pullin qualified as far back as 1850 from St. Bartholomew's Hospital; he became M.D. St. Andrews in 1862 and took the Fellowship of the Royal College of Surgeons of Edinburgh in 1882. He was Medical Officer of Health for Sidmouth, certifying factory surgeon, and surgeon to the Sidmouth Dispensary. During the cholera epidemic of 1849 Dr. Pullin acted as medical officer to the Government Board of Health at Portsmouth, and in 1850 he was already surgeon to Christ's Hospital. His activities found vent in the interest he took in many learned societies and in numerous contributions to the medical journals.

The death is announced of Dr. William Millington, of Wolverhampton, in his ninety-first year. He was one of the oldest medical practitioners in England, and nearly the oldest member of the Royal College of Physicians of London, his membership dating from 1859. He graduated M.D. at the University of Edinburgh in 1851, obtaining the gold medal for a thesis on "The Pathology of Pus in the Blood." Dr. Millington was consulting physician to the Wolverhampton and Staffordshire General Hospital, with which institution he was for very many years closely connected on the medical side. He was also physician to the Wolverhampton Orphanage.

